# Control of multidrug resistant (MDR) *Acinetobacter baumanii* outbreak in a pediatric oncology unit by optimizing infection prevention (IP) and control measures

**DOI:** 10.1186/1753-6561-5-S6-P94

**Published:** 2011-06-29

**Authors:** MA Melgar, RE De Leon, E Asturias, F Antillon-Klussman, M Caniza

**Affiliations:** 1 Infectious Diseases, Unidad Nacional de Oncologia Pediatrica, Guatemala, Guatemala; 2 Infection Contrl, Unidad Nacional de Oncologia Pediatrica, Guatemala, Guatemala; 3 Medial Director, Unidad Nacional de Oncologia Pediatrica, Guatemala, Guatemala; 4 Infectious Diseases/International Outreach Program, StJude Childrens Research Hospital, Memphis TN, USA

## Introduction / objectives

*Acinetobacter baumanii* is reconized as an important MDR pathogen. We present results of our effort to control a recent outbreak in our ICU

## Methods

Our hospital is a 40 bed pediatric oncology unit. We defined our cases as any patient in the ICU positive for MDR *A.baumanii* from September 1 - October 31, 2010. Our intervention consisted of enforcing contact isolation (gloves and gown) for positive patients, update guidelines for airway management, and education for all ICU personnel.

## Results

In the study period, 4 patients had tracheal aspirate (TA) positive for MDR *A.baumanii* (all on mechanical ventilation), one patient had positive catheter tip and blood cultures. The attack rate was 12,8% and the ventilator associated pneumonia rate rose from 20,30 to 34,96 per 1000 ventilator days. The first three cases occurred from September 13 to 20 all of them in TA. Contact precautions were initiated for positive patients. After a 20 day event free period, a patient developed signs of infection and had culture with *A.baumanii*; IP and control education was given to the respiratory therapy team and ICU staff including hand hygiene promotion and airway management. Two additional cases occurred on October 18 and 20, since there have been no new cases.

## Conclusion

Expedited basic IP and control measures implementation such as education, hand hygiene promotion, isolation precautions and adequate airway management controlled the outbreak of MDR *A. baumanii.* Universal IP and control education is key for successful outbreak containment.

## Disclosure of interest

None declared.

